# Retrospective study on the distribution of pathogens, antibiotic resistance patterns, and risk factors of multidrug-resistant organism infections in patients with type 2 diabetic foot ulcers

**DOI:** 10.1097/MD.0000000000045888

**Published:** 2026-01-30

**Authors:** Jialin Huang, Dan Huang, Daixiong Tian

**Affiliations:** a Medical Cosmetology and Plastic Surgery, Burn Surgery Department, The Central Hospital of Enshi Tujia and Miao Autonomous Prefecture, Enshi, Hubei Province, China.

**Keywords:** antibiotic resistance, diabetic foot ulcer, logistic regression analysis, multidrug-resistant organisms, pathogen distribution, risk factors

## Abstract

Multidrug-resistant organism (MDRO) infections are increasingly prevalent in patients with type 2 diabetic foot ulcers (DFUs), posing substantial challenges to treatment efficacy and clinical outcomes. Understanding the risk factors and epidemiological characteristics of MDRO infections is critical for formulating effective antimicrobial strategies. This single-center retrospective study included 247 patients hospitalized with DFUs between January 2022 and December 2024. Based on microbiological findings, patients were classified into an MDRO group (n = 102) and a non-MDRO group (n = 145). Data on demographics, pathogen distribution, antibiotic resistance profiles, and clinical outcomes were collected. Logistic regression analysis was performed to identify independent risk factors for MDRO infection, and clinical outcomes were compared between the 2 groups. Patients in the MDRO group had a longer duration of diabetes and higher HbA1c levels, along with a significantly greater proportion of Wagner grade ≥ 3 ulcers and peripheral arterial disease (all *P* < .05). A total of 268 pathogenic strains were isolated, predominantly Gram-negative bacteria (62.3%), with *Pseudomonas aeruginosa*, *Escherichia coli*, and *Acinetobacter baumannii* being the most common. The leading MDROs were extended-spectrum β-lactamase-producing *E. coli* (14.6%) and carbapenem-resistant *A. baumannii* (11.2%). Multivariate analysis identified Wagner grade ≥ 3 (odds ratio [OR] = 2.31), prior use of broad-spectrum antibiotics (OR = 2.87), and peripheral arterial disease (OR = 1.96) as independent risk factors for MDRO infection. Compared with the non-MDRO group, the MDRO group had significantly higher rates of treatment failure (34.3% vs 18.6%), readmission (21.6% vs 10.2%), and greater antibiotic use, both in number and duration (all *P* < .05). Higher Wagner grades were also associated with increased rates of MDRO and mixed infections (*P* < .001). MDRO infections are common among patients with DFUs, with Gram-negative bacteria as the predominant pathogens. Higher Wagner grade, prior antibiotic exposure, and peripheral arterial disease are independent risk factors. MDRO infections impose a greater treatment burden and are linked to poorer prognoses. Early identification of high-risk patients, coupled with optimized infection control and antimicrobial stewardship, is essential to improving clinical outcomes.

## 1. Introduction

Diabetic foot ulcer (DFU) is among the most common and serious chronic complications of diabetes mellitus. Globally, an estimated 15% to 25% of individuals with diabetes will develop a foot ulcer during their lifetime.^[1,2]^ According to the International Diabetes Federation (IDF), DFU is now a leading cause of diabetes-related hospitalizations, lower-limb amputations, and even mortality.^[3–5]^ The pathogenesis of DFU is multifactorial, involving peripheral neuropathy, peripheral arterial disease, immune dysfunction, and infection.^[6,7]^ Among these factors, infection is recognized as a major driver of disease progression and a key contributor to the risk of amputation. In particular, infections caused by multidrug-resistant organisms (MDROs) have emerged as a critical determinant of treatment success and overall prognosis.^[8,9]^

MDROs are generally defined as pathogenic microorganisms resistant to at least 3 classes of antimicrobial agents, and include extended-spectrum β-lactamase (ESBL)-producing Gram-negative bacteria, carbapenem-resistant *Acinetobacter baumannii*, and methicillin-resistant *Staphylococcus aureus* (MRSA).^[10–13]^ In recent years, the incidence of MDRO infections has risen sharply, driven by factors such as the overuse of broad-spectrum antibiotics, increasing comorbidity burden, and prolonged hospitalization. This trend is particularly pronounced in chronic wounds like DFUs, where the detection rate of MDROs continues to increase annually.^[14]^ Numerous studies have shown that MDRO infections in DFU patients markedly reduce treatment success, prolong hospital stays, and increase readmission rates, healthcare costs, and the risk of amputation.^[15–18]^ Consequently, a thorough understanding of MDRO pathogen distribution, antimicrobial resistance patterns, and associated risk factors in DFU patients is essential to inform precision treatment, antimicrobial stewardship, and infection control measures.

Previous research has identified both Gram-negative bacteria, such as *P. aeruginosa, Escherichia coli*, and *A. baumannii*, and Gram-positive bacteria, such as *S. aureus* and Enterococcus species, as the predominant pathogens in DFU infections.^[19]^ However, pathogen spectra and resistance profiles vary widely depending on geographic region, ulcer severity, comorbidities, and antibiotic usage practices. Although several studies, both domestic and international, have examined the risk factors for MDRO infections in DFU patients, many have been constrained by small sample sizes and limited scope, with a notable paucity of comprehensive epidemiological data from Eastern China.^[20]^ Enshi Tujia and Miao Autonomous Prefecture is located in western Hubei Province, China, characterized by a mixed urban–rural population and relatively limited access to advanced wound care resources. Regional differences in climate, socioeconomic status, and antibiotic usage practices may contribute to variations in pathogen distribution and antimicrobial resistance patterns compared to other parts of China. However, epidemiological data on MDRO infections in diabetic foot ulcers (DFUs) from this region remain scarce. Therefore, a systematic analysis of the clinical and microbiological features of hospitalized DFU patients in this region is of high practical and clinical relevance.

This study retrospectively analyzed clinical data from patients with type 2 diabetes and DFU who were hospitalized at a tertiary hospital in Jiangsu Province between January 2022 and December 2024. Collected variables included demographic characteristics, clinical parameters, Wagner ulcer grade, pathogen culture results, and antimicrobial susceptibility profiles. Based on microbiological findings, patients were classified into MDRO and non-MDRO groups, and their baseline characteristics were compared. Logistic regression analysis was performed to identify independent risk factors for MDRO infection and to assess its impact on clinical outcomes. We hypothesized that the prevalence, pathogen spectrum, and antimicrobial resistance patterns of MDRO infections in DFU patients in this region would differ from those reported elsewhere, and that certain clinical factors, such as ulcer severity and comorbidities, would be independently associated with MDRO infection. By systematically characterizing the epidemiological patterns and determinants of MDRO infections in hospitalized DFU patients, this study aims to provide evidence to support early identification of high-risk individuals and optimization of empirical antimicrobial treatment strategies in clinical practice.

## 2. Method

### 2.1. Study design and participants

This study was a single-center, retrospective case-control study. The study population consisted of patients with type 2 DFUs who were hospitalized in our institution between January 2022 and December 2024. Inclusion criteria were as follows: diagnosis of type 2 diabetes mellitus in accordance with the 2020 Chinese guidelines; presence of an infected foot ulcer; and completion of microbiological culture and antimicrobial susceptibility testing during hospitalization. Exclusion criteria included concurrent malignancy or immunodeficiency disorders; foot infections caused by trauma, burns, or other non-diabetes-related factors; and incomplete hospitalization records.

### 2.2. Grouping and sample size calculation

Patients were divided into 2 groups based on the microbiological findings of wound cultures: an MDRO infection group and a non-MDRO group. The definition of MDROs followed the 2022 Chinese Guidelines for Clinical Use of Antimicrobial Agents. Preliminary data from a pilot study showed that the treatment failure rate was approximately 35% in the MDRO group and 20% in the non-MDRO group. Assuming a significance level of α = 0.05 and a power (1 − β) of 0.8, the minimum required sample size was estimated to be 228 cases using PASS 15.0 software. A total of 247 patients were ultimately included in the study, comprising 102 in the MDRO group and 145 in the non-MDRO group, meeting the statistical power requirement.

### 2.3. Clinical and laboratory data collection

Demographic data, diabetes duration, Wagner classification, peripheral arterial disease status, and prior antibiotic use were retrieved from the hospital’s electronic medical record system. Additional clinical indicators such as fasting plasma glucose, HbA1c, blood pressure, and body mass index were also collected. Upon admission, all patients underwent standardized wound debridement and bacterial culture. Bacterial identification and antimicrobial susceptibility testing were performed using the VITEK-2 automated system. Major MDROs included ESBL-producing *E. coli*, carbapenem-resistant *A. baumannii*, and MRSA.

### 2.4. Statistical analysis

Data were analyzed using SPSS version 26.0. Continuous variables were tested for normality and presented as mean ± standard deviation; comparisons between groups were made using independent-sample *t*-tests. Categorical variables were expressed as frequencies (percentages) and compared using the chi-square (χ²) test. The proportion of missing data for all collected variable data is <5%, and mean and mode are used for filling (applicable to continuous variables and categorical variables respectively). Logistic regression analysis was conducted to identify independent risk factors for MDRO infection. Variables included in the model were diabetes duration, HbA1c level, Wagner grade, prior use of antibiotics, and presence of peripheral arterial disease. A stepwise regression approach was used for multivariate analysis, with statistical significance set at *P* < .05.

### 2.5. Ethical statement

This study was approved by the The Central Hospital Of Enshi Tujia and Miao Autonomous Prefecture Ethics Committee. As a retrospective analysis, the requirement for informed consent was waived. All data were handled in strict accordance with protocols for patient confidentiality and privacy protection.

## 3. Result

### 3.1. Patient enrollment and baseline characteristics comparison

A total of 247 patients with type 2 DFUs who were hospitalized at our institution between January 2022 and December 2024 were included in this study. Based on the presence or absence of MDRO infection, patients were classified into the MDRO group (n = 102) and the non-MDRO group (n = 145). Baseline characteristics were compared between the 2 groups. The MDRO group had a significantly longer duration of diabetes than the non-MDRO group (12.7 ± 6.5 years vs 9.3 ± 5.1 years, *P* < .001) and significantly higher hemoglobin A1c (HbA1c) levels (9.6 ± 1.3% vs 8.7 ± 1.2%, *P* = .002). Systolic blood pressure was also slightly higher in the MDRO group (142.6 ± 18.7 mm Hg vs 138.4 ± 17.9 mm Hg, *P* = .049). The proportion of patients with Wagner grade ≥ 3 ulcers was markedly higher in the MDRO group compared with the non-MDRO group (67.6% vs 41.4%, *P* < .001), as was the prevalence of peripheral arterial disease (58.8% vs 36.6%, *P* = .001). No statistically significant differences were observed between the groups in age, sex, body mass index, diastolic blood pressure, heart rate, height, or weight (all *P* > .05). Detailed comparisons are shown in Table 1.

**Table 1:** Baseline characteristics comparison table.

Variable	MDRO group (n = 102)	Non-MDRO group (n = 145)	χ²/*t* value	*P* value
Age (yr)	66.2 ± 9.5	64.8 ± 10.1	1.07	.284
Sex (male, n)	64 (62.7%)	88 (60.7%)	0.11	.741
Duration of diabetes (yr)	12.7 ± 6.5	9.3 ± 5.1	4.72	<.001*
HbA1c (%)	9.6 ± 1.3	8.7 ± 1.2	3.19	.002*
BMI (kg/m²)	25.8 ± 3.2	25.4 ± 3.5	0.71	.478
Systolic BP (mm Hg)	142.6 ± 18.7	138.4 ± 17.9	1.98	.049*
Diastolic BP (mm Hg)	83.4 ± 10.5	81.2 ± 9.7	1.81	.072
Heart rate (beats/min)	86.1 ± 11.6	84.6 ± 10.9	1	.318
Height (cm)	165.3 ± 7.9	166.1 ± 8.3	0.58	.563
Weight (kg)	70.7 ± 11.4	71.2 ± 12.1	0.4	.692
Wagner grade ≥ 3 (n)	69 (67.6%)	60 (41.4%)	14.06	<.001*
Peripheral arterial disease (n)	60 (58.8%)	53 (36.6%)	10.43	.001*

### 3.2. Distribution of pathogens

**Figure 1.**
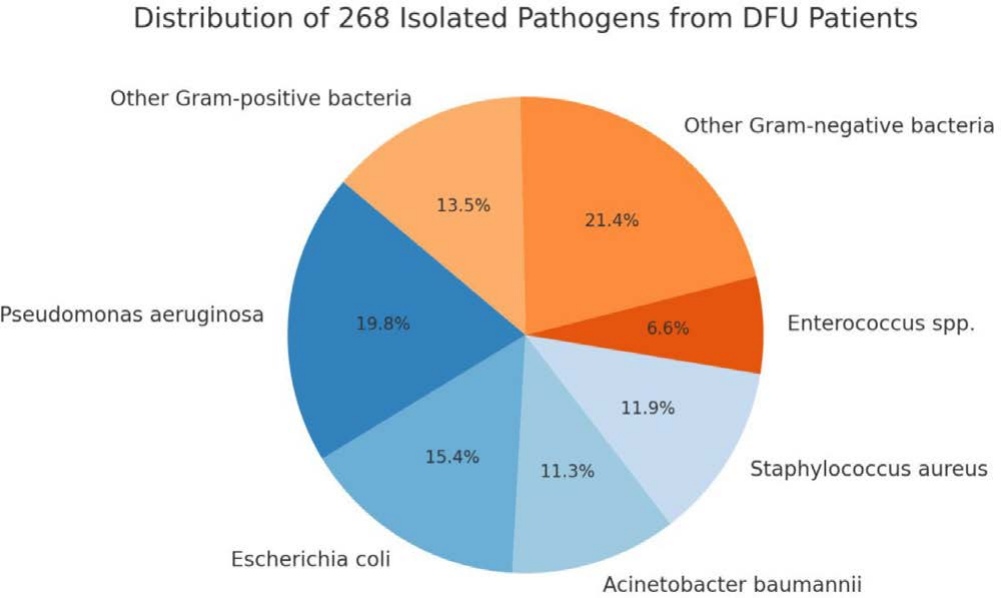
Distribution of 268 isolated pathogens from DFU patients. DFU = diabetic foot ulcer.

A total of 268 pathogenic strains were isolated from wound specimens of 247 patients with type 2 DFUs included in this study. Analysis of bacterial species showed that Gram-negative bacteria predominated, accounting for 167 isolates (62.3%), while Gram-positive bacteria comprised 101 isolates (37.7%). Among all isolates, *P. aeruginosa* was the most frequently detected organism (23.5%, 63/268), followed by *E. coli* (18.3%, 49/268) and *A. baumannii* (13.4%, 36/268). Among Gram-positive bacteria, the most common pathogens were *S. aureus* (14.2%, 38/268) and Enterococcus species (7.8%, 21/268). Further analysis revealed that, among MDROs, the most prevalent were ESBL-producing *E. coli* (14.6%) and carbapenem-resistant *A. baumannii* (11.2%). These findings indicate a high prevalence of antibiotic-resistant infections in DFU patients, underscoring the urgent need for strengthened antimicrobial stewardship and targeted infection control measures. Figure 1 illustrates the distribution of different pathogen types, clearly demonstrating the predominance of Gram-negative bacteria in DFU-associated infections.

### 3.3. Antimicrobial resistance profiles

**Figure 2.**
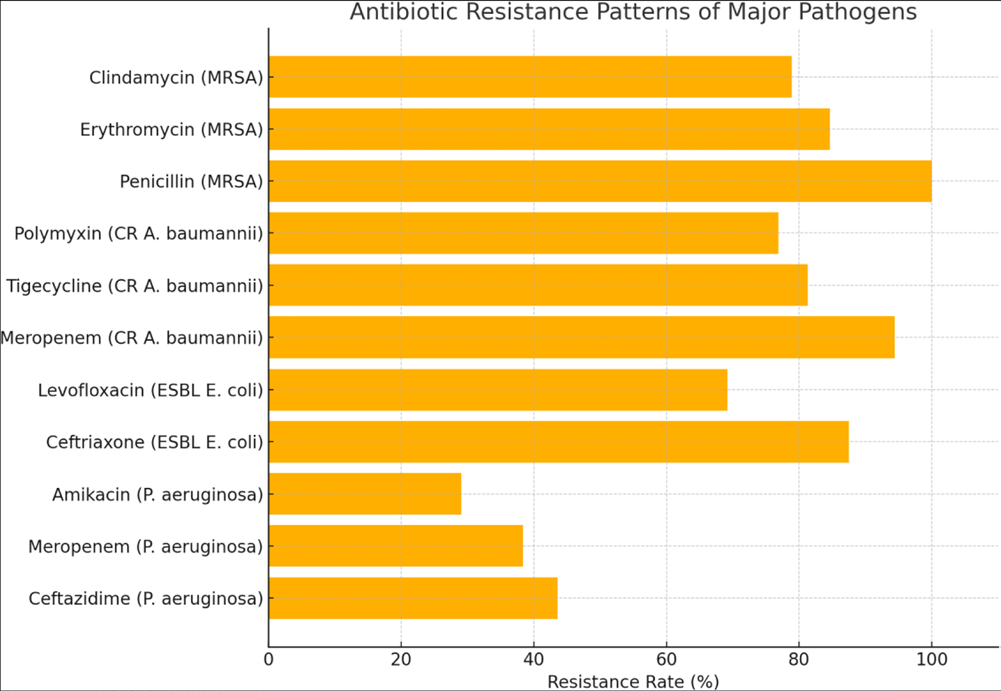
Antibiotic resistance patterns of major pathogens.

The antimicrobial susceptibility results of the major pathogens isolated in this study were statistically analyzed, revealing that several strains exhibited marked multidrug resistance, posing considerable challenges to clinical treatment. *P. aeruginosa* demonstrated high resistance rates to several commonly used antibiotics: 43.6% to ceftazidime, 38.4% to meropenem, and 29.1% to amikacin. These findings indicate that this organism has developed considerable resistance to both β-lactam and aminoglycoside antibiotics, suggesting that empirical treatment regimens should be selected with caution. ESBL-producing *E. coli* showed alarmingly high resistance rates to ceftriaxone (87.5%) and levofloxacin (69.2%), indicating extensive resistance to third-generation cephalosporins and fluoroquinolones. This raises concerns about the limited effectiveness of conventional antimicrobial regimens.Carbapenem-resistant *A. baumannii* exhibited particularly severe resistance profiles, with resistance rates of 94.4% to meropenem, 81.3% to tigecycline, and 76.9% to polymyxin. These results suggest that this pathogen is virtually insensitive to standard antimicrobial therapies, and highly individualized treatment strategies are urgently required. MRSA also demonstrated significant resistance, with resistance rates of 100% to penicillin, 84.6% to erythromycin, and 78.9% to clindamycin. These data highlight the importance of considering resistance backgrounds when treating Gram-positive infections and avoiding blind use of traditional antibiotics. Figure 2 presents the resistance profiles of the major pathogens to representative antibiotics, visually emphasizing the high detection rate of multidrug-resistant strains among patients with type 2 DFUs. These findings provide essential guidance for the rational selection of antimicrobial agents in clinical practice.

### 3.4. Risk factor analysis for MDRO infection

**Figure 3.**
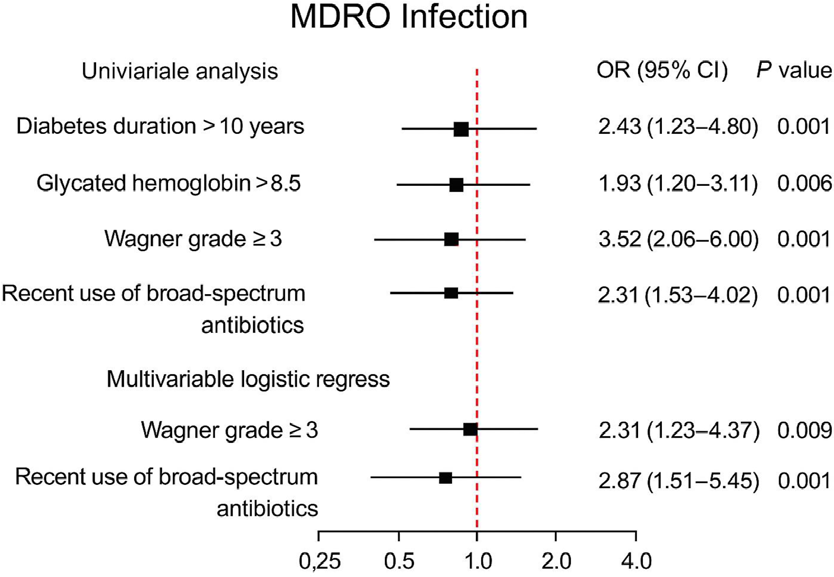
Forest plot of univariable and multivariable logistic regression analyses for MDRO infection in patients with type 2 diabetic foot ulcers. MDRO = multidrug-resistant organism.

To further identify factors associated with MDRO infections in patients with type 2 DFUs, both univariate and multivariate logistic regression analyses were performed. Univariate analysis showed significant associations between MDRO infection and the following variables (all *P* < .05): diabetes duration > 10 years, hemoglobin A1c (HbA1c) > 8.5%, Wagner grade ≥ 3, recent broad-spectrum antibiotic use, and presence of peripheral arterial disease. These variables were then included in a multivariate logistic regression model. The results indicated that Wagner grade ≥ 3 was independently associated with MDRO infection (odds ratio [OR] = 2.31, 95% confidence interval [CI]: 1.23–4.37, *P* = .009), suggesting that more severe ulcers are linked to a higher likelihood of MDRO infection. A recent history of broad-spectrum antibiotic use was strongly associated with an increased risk of infection (OR = 2.87, 95% CI: 1.51–5.45, *P* = .001), possibly due to antibiotic-induced disruption of the normal microbiota and selective growth of resistant organisms.Peripheral arterial disease was independently associated with MDRO infection (OR = 1.96, 95% CI: 1.02–3.77, *P* = .043), underscoring the association between impaired blood supply and heightened susceptibility to infection. Detailed results are illustrated in Figure 3.

### 3.5. Impact of MDRO infection on clinical outcomes

This study further evaluated the impact of MDRO infections on clinical outcomes in patients with type 2 DFUs. The results showed that patients in the MDRO group had markedly poorer outcomes across several key clinical indicators. The treatment failure rate was 34.3% in the MDRO group, significantly higher than 18.6% in the non-MDRO group (*P* = .006). Likewise, the readmission rate was markedly higher in the MDRO group (21.6% vs 10.2%, *P* = .013). In terms of antimicrobial therapy, patients with MDRO infections received a greater variety of antibiotic agents (3.4 ± 1.1 vs 2.2 ± 0.8, *P* < .001), and their treatment duration was also significantly longer (16.2 ± 5.7 days vs 11.3 ± 4.6 days, *P* < .001). These findings indicate that MDRO infections are associated with a substantially greater therapeutic burden and are linked to poorer prognoses in DFU patients. Detailed comparisons of these outcome indicators are presented in Table 2.

**Table 2:** Statistical table of prognostic impact of MDRO infection.

Variable	MDRO group	Non-MDRO group	*P* value
Treatment failure rate	34.30%	18.60%	.006
Readmission rate	21.60%	10.20%	.013
Number of antibiotic types	3.4 ± 1.1	2.2 ± 0.8	<.001
Duration of antibiotic therapy (d)	16.2 ± 5.7	11.3 ± 4.6	<.001

MDRO = multidrug-resistant organism.

### 3.6. Relationship between wound bacterial distribution and Wagner grade

This study further examined the relationship between wound bacterial distribution and Wagner classification in patients with DFUs. The proportion of MDRO infections increased progressively with higher Wagner grades, from 14.3% in grade 1 to 29.1% in grade 2 and 52.8% in grade 3 or above. A trend test confirmed a statistically significant correlation (*P* < .001), indicating a positive association between ulcer severity and the likelihood of MDRO infection.

**Figure 4.**
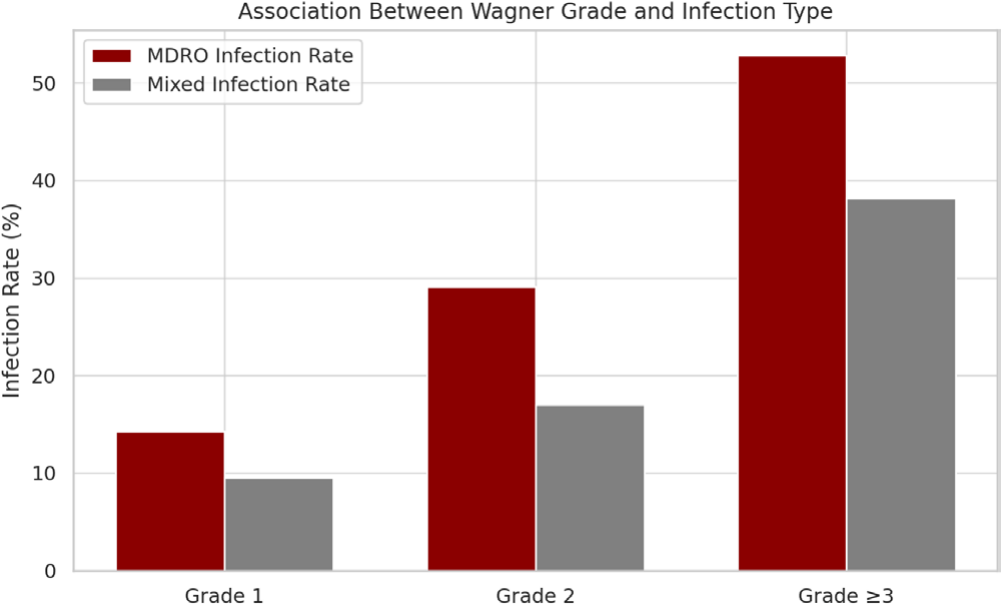
Association between Wagner grade and infection type.

Furthermore, the prevalence of mixed infections also rose with increasing Wagner grades, particularly for complex infections involving both Gram-positive and Gram-negative bacteria. The rates of mixed infection were 9.5% for grade 1, 17.0% for grade 2, and 38.2% for grade 3 or above. These findings, illustrated in Figure 4, underscore the importance of considering ulcer severity when predicting pathogen complexity and formulating empirical antimicrobial strategies.

## 4. Discussion

The increasing prevalence of MDRO infections in patients with DFUs has become a growing public health concern, significantly affecting treatment efficacy and clinical prognosis.^[21]^ Due to underlying immune dysfunction, peripheral neuropathy, and peripheral arterial disease, DFU patients are highly susceptible to infections, which often progress to chronic refractory wounds or even sepsis.^[22]^ The presence of MDROs further complicates antimicrobial therapy, increases the burden of antibiotic use, prolongs hospitalization, and markedly elevates the risks of readmission and amputation.^[23]^ Therefore, identifying the risk factors for MDRO infections and understanding pathogen distribution and resistance profiles are essential for guiding precision treatment and infection control.

This study retrospectively analyzed clinical data from 247 hospitalized DFU patients between January 2022 and December 2024, aiming to explore the epidemiological features and clinical impact of MDRO infections. The findings have strong practical relevance and provide guidance for clinical decision-making.

Comparison of baseline characteristics revealed that patients with MDRO infections had significantly longer durations of diabetes, higher HbA1c levels, and elevated systolic blood pressure, suggesting more severe metabolic dysfunction. Chronic hyperglycemia may promote bacterial colonization and infection, consistent with previous studies reporting an association between prolonged diabetes duration and MDRO infection, as well as impaired tissue perfusion and immune defense caused by long-standing hyperglycemia.^[24]^ Furthermore, a higher prevalence of Wagner grade ≥ 3 ulcers and peripheral arterial disease was observed in the MDRO group, indicating that severe wounds and localized ischemia may provide a favorable environment for colonization and spread of resistant organisms.

In terms of pathogen distribution, a total of 268 strains were isolated, with Gram-negative bacteria being the predominant group. *Pseudomonas aeruginosa* and *E. coli* were the most frequently detected species. The major MDRO types included ESBL-producing *E. coli* and carbapenem-resistant *A. baumannii*. These results are consistent with bacterial spectra reported in multicenter studies across China, confirming the dominance of Gram-negative bacilli in DFU infections.^[13]^ The high detection rate of MDROs, particularly in patients with severe ulcers, underscores the need for broad-spectrum pathogen coverage in empirical antimicrobial therapy and reinforces the importance of routine culture and susceptibility testing.

The predominance of Gram-negative bacteria in our cohort may be explained by several factors. First, DFUs often present as chronic wounds with abundant exudate and tissue necrosis, creating a moist, nutrient-rich environment that favors the colonization and proliferation of Gram-negative bacilli such as *P. aeruginosa* and *E. coli*. Second, many patients in this study had a history of broad-spectrum antibiotic exposure, which may have selectively suppressed Gram-positive flora and facilitated the dominance of Gram-negative organisms. Third, regional characteristics of Jiangsu Province, including climate, hygiene practices, and patterns of healthcare-associated pathogen transmission, may also contribute to the higher detection rates of Gram-negative species. Similar findings have been reported in other studies from China and Southeast Asia, suggesting that Gram-negative predominance may be a common feature in regions with comparable environmental and healthcare contexts.

Antibiotic resistance profiling further underscores the challenge of current infection management. *P. aeruginosa* showed high resistance to ceftazidime, meropenem, and amikacin, while *A. baumannii* exhibited extensive resistance to nearly all standard antimicrobial agents. The notably high resistance rates of carbapenem-resistant *A. baumannii* and MRSA raise serious concerns about the effectiveness of existing treatment options. This highlights the importance of tailoring antimicrobial regimens based on local resistance patterns and avoiding indiscriminate use of broad-spectrum antibiotics to mitigate further resistance development.

Logistic regression analysis identified Wagner grade ≥ 3, recent use of broad-spectrum antibiotics, and peripheral arterial disease as independent risk factors for MDRO infection. Notably, this study is among the first to systematically validate these independent associations in a single-center DFU cohort in China, providing novel insights with implications for wider clinical practice.

MDRO infection had a significant negative impact on patient prognosis. The MDRO group exhibited substantially higher treatment failure and readmission rates, along with greater antibiotic consumption and longer treatment durations. These findings underscore the increased disease burden imposed by MDROs and emphasize the importance of early detection and effective infection control to improve DFU outcomes.

Furthermore, we found a strong correlation between ulcer severity and infection complexity. Higher Wagner grades were associated with increased rates of MDRO and mixed infections, especially those involving both Gram-positive and Gram-negative organisms. This may be attributed to tissue necrosis, heavy exudation, and compromised barrier function in advanced ulcers. These findings suggest that routine culture testing and heightened vigilance for polymicrobial infections are warranted in patients with higher Wagner grades.

Based on the observed antimicrobial resistance patterns, we propose the following empirical anti-infective strategies for DFU patients in similar settings. For infections suspected to be caused by Gram-negative organisms such as *P. aeruginosa* or ESBL-producing *E. coli*, initial empirical therapy may include β-lactam/β-lactamase inhibitor combinations (e.g., piperacillin-tazobactam) or carbapenems in severe cases. For suspected infections with *A. baumannii*, given the high resistance to carbapenems and even polymyxins, individualized treatment based on susceptibility results is essential, and combination therapy may be considered. For Gram-positive organisms, especially MRSA, empirical therapy with linezolid or vancomycin may be warranted in patients with Wagner grade ≥ 3 ulcers. De-escalation should be guided by culture and sensitivity results as soon as available, and empirical regimens should be adjusted based on local antibiogram data. These recommendations aim to enhance initial treatment effectiveness, reduce inappropriate antibiotic use, and minimize the risk of further resistance.

In conclusion, this study provides a comprehensive analysis of the epidemiological features, risk factors, and clinical consequences of MDRO infections in DFU patients, filling a critical gap in domestic research. The results have meaningful clinical implications. We recommend that clinicians strengthen early identification of high-risk patients, optimize antibiotic stewardship strategies, and prioritize assessment of Wagner grade and peripheral arterial disease to enable precise management of DFU infections and mitigate the risk of MDRO-associated adverse outcomes. Future studies should consider prospective multicenter cohort designs and molecular epidemiological approaches to further elucidate the transmission mechanisms of MDROs in DFUs and their interaction with host factors.

## 5. Limitations

This study has several limitations. First, it was conducted in a single tertiary hospital, and the pathogen spectrum and resistance patterns observed may not be representative of other regions or healthcare settings. Second, as a retrospective study, it is subject to inherent biases such as incomplete data capture, unmeasured confounding, and potential selection bias, despite efforts to adjust for multiple variables. Third, although bacterial culture and antimicrobial susceptibility testing were performed using standardized laboratory procedures within our institution, the results may still be influenced by the detection techniques employed, and variations in methodology across institutions could affect comparability. Fourth, while we reported the incidence of mixed infections, we did not conduct a detailed analysis of their prognostic impact, such as treatment failure, length of hospital stay, amputation rates, or readmission rates; this warrants further investigation. Fifth, molecular typing and resistance gene profiling of the isolates were not performed, which could provide deeper insights into the epidemiology and transmission dynamics of MDROs. Finally, due to the observational design, residual confounding cannot be fully excluded despite multivariate adjustment.

Future multicenter prospective studies should evaluate the prognostic significance of mixed infections, explore molecular resistance mechanisms, and validate region-specific empirical antimicrobial strategies based on continuous surveillance of pathogen distribution and resistance trends.

## 6. Conclusion

This study systematically reviewed and analyzed the epidemiological characteristics, associated risk factors, and clinical impact of MDRO infections in patients with type 2 DFUs. The results demonstrated a high prevalence of MDRO infections in this population, which were significantly associated with longer diabetes duration, poor glycemic control, higher Wagner grades, and peripheral arterial disease.The predominant resistant pathogens were ESBL-producing *E. coli* and carbapenem-resistant *A. baumannii*, both of which exhibited broad-spectrum resistance, posing considerable challenges to clinical management. Logistic regression analysis identified Wagner grade ≥ 3, a history of broad-spectrum antibiotic use, and the presence of peripheral arterial disease as independent risk factors for MDRO infection. MDRO infection was found to significantly impair clinical outcomes in DFU patients, as evidenced by higher treatment failure and readmission rates, along with an increased burden of antimicrobial use.Therefore, it is crucial to strengthen the identification and stratified management of high-risk patients, implement standardized antimicrobial stewardship strategies, and optimize infection control pathways in order to improve treatment efficacy and reduce the risk of MDRO-related complications.

## Author contributions

**Conceptualization:** Daixiong Tian.

**Data curation:** Daixiong Tian.

**Investigation:** Daixiong Tian.

**Methodology:** Daixiong Tian.

**Validation:** Daixiong Tian.

**Writing – original draft:** Daixiong Tian.

**Writing – review & editing:** Daixiong Tian.
